# Recovery from Wound of the Head, with Loss of Substance of the Brain

**Published:** 1845-11

**Authors:** 


*Recovery from Wound of the Head, with loss of substance of the Brain.* By M. Rouelle.—An individual was struck over the head, in a quarrel, with a heavy baton, and instantly fell. M. Rouelle, who saw him within a quarter of an hour after the accident, recognised a comminuted fracture on the superior part of the skull. A portion of the cerebral substance, about the size of an apricot stone, had escaped through a wound situated over the union between the frontal and right parietal bone. At this time the person did not present any symptoms of intellectual lesion; he answered readily and collectedly to questions addressed to him relative to the seat of the pain, &c. The whole left side of the body, however, was paralyzed; the mouth, however, was not drawn to one side. Notwithstanding the gravity of the accident, and the occasional severe symptoms which occurred during the course of the treatment, a cure was effected. By the end of six months the patient had quite recovered the use of the leg; the arm, however, still remained paralyzed.—*Ibid, from Comp. Ren. des Sean. de l'Acad. Roy. des Sciences.*

